# The immunogenicity and tissue reactivity of *Mycobacterium avium* subsp *paratuberculosis* inactivated whole cell vaccine is dependent on the adjuvant used

**DOI:** 10.1016/j.heliyon.2019.e01911

**Published:** 2019-06-18

**Authors:** D.J. Begg, O. Dhungyel, A. Naddi, N.K. Dhand, K.M. Plain, K. de Silva, A.C. Purdie, R.J. Whittington

**Affiliations:** School of Veterinary Science, Faculty of Science, The University of Sydney, NSW, Australia

**Keywords:** Immunology, Microbiology

## Abstract

Johne's disease (JD) is a chronic enteritis caused by *Mycobacterium avium* subspecies *paratuberculosis* (MAP). Current commercial vaccines are effective in reducing the occurrence of clinical disease although vaccinated animals can still become infected and transmit MAP. Many vaccinated sheep develop severe injection site lesions. In this study a range of adjuvants (Montanide^**TM**^ ISA 50V, ISA 50V2, ISA 61VG, ISA 70 M VG, ISA 71 VG, ISA 201 VG and Gel 01 PR) formulated with heat-killed MAP were tested to determine the incidence of injection site lesions and the types of immune profiles generated in sheep. All the novel formulations produced fewer injection site lesions than a commercial vaccine (Gudair®). The immune profiles of the sheep differed between treatment groups, with the strength of the antibody and cell mediated immune responses being dependant on the adjuvant used. One of the novel vaccines resulted in a reduced IFN-γ immune response when a second “booster” dose was administered. These findings have significance for JD vaccine development because it may be possible to uncouple protective immunity from excessive tissue reactivity, and apparently poorly immunogenic antigens may be re-examined to determine if an appropriate immune profile can be established using different adjuvants. It may also be possible to formulate vaccines that produce targeted immunological profiles suited to protection against other pathogens, i.e. those for which a bias towards cellular or humoral immunity would be advantageous based on understanding of pathogenesis.

## 1 Introduction

Johne's disease (JD) is a chronic enteritis caused by *Mycobacterium avium* subspecies *paratuberculosis* (MAP). Typically, it is spread within and between herds/flocks of ruminants by the faecal-oral route. The disease results in weight loss and mortality and can cause significant economic impact for farmers [1]. While treatment for JD is not feasible, vaccination is being used as one of the key control measures, especially in sheep [2].

Vaccination against JD has been in use since the 1920s with mixed success. Current commercial vaccines are effective in reducing clinical disease occurrence by up to 90% giving farmers an important disease control tool [3]. Vaccinated animals can still become infected and shed MAP in their faeces [3]. The current vaccines, including the Gudair® vaccine, are based on killed whole MAP cells mixed with an oil adjuvant [3]. One of the major concerns with these vaccines is their tendency to result in lesions at the site of injection in a proportion of animals [4]. Also of concern to users of these vaccines is human safety, because recovery from accidental self-injection may take months and require multiple medical treatments, often involving surgical intervention [5].

The adjuvant portion of a vaccine plays an import role in its efficacy [6]. In the case of JD vaccines these have been mineral oils [3, 7] although their exact composition is not disclosed due to commercial considerations. Mineral oil adjuvants, when mixed with whole mycobacterial cells, can often lead to injection site responses similar to those seen when using Freund's complete adjuvant [5]. Therefore, it is hardly surprising that injection site lesions in sheep are prevalent after vaccination with the current commercial JD vaccines.

Recently, highly refined mineral oil emulsion adjuvants have become available. They are of several types: water in oil (W/O), water in oil in water (W/O/W) and oil in water (O/W) [6]. A newly developed killed whole cell vaccine for JD, Silirium®, uses a highly refined mineral oil adjuvant which should result in fewer injection site lesions than Gudair®, but like Gudair® it does not prevent infection [3].

Most recent novel vaccination studies against MAP infection have examined one or two adjuvants, generally from different adjuvant classes such as alum and saponin [8, 9, 10, 11]. In a study of the immunogenicity of a recombinant *M. bovis* antigen in cattle it was noticed that different classes of adjuvants, mineral oils and cationic liposome-based formulations, resulted in different immune response profiles [12]. The mineral oil-based adjuvants resulted in an effector and a central memory response while the cationic, liposome-based formulations resulted in strong central memory responses. Differences in the immune response were also observed amongst the several mineral oil adjuvants used [12].

In this study, we aimed to characterise the immunological responses to MAP antigens associated with a range of adjuvants. The immunogenicity of formulations containing heat killed MAP mixed with one of seven different adjuvants (mineral oil or polymeric gel) administered to sheep with or without a booster dose was examined.

## 2 Methods

### 2.1 Animals

Ninety Merino wethers aged 24–36 months were sourced from a flock in Armidale, New South Wales, an area that has no prior history of JD. Absence of JD was confirmed through repeated whole flock faecal tests and antibody enzyme linked immunosorbent assays (ELISA) [13]. The animals were moved to a JD-free quarantine farm at the University of Sydney Camden and maintained under conventional Australian sheep farming conditions by grazing on open pasture.

### 2.2 Ethical considerations

All animal experiments were conducted with the approval of the University of Sydney Animal Ethics Committee.

### 2.3 Treatment groups

Sheep were allocated into 18 groups, with five sheep per group. The first eight groups were allocated for a single dose of the novel vaccines (Table 1). The remaining eight groups were allocated a primary and a booster dose of the novel vaccines. The booster dose was administered 4 weeks after the primary dose. One group, the positive control, was given the commercially available vaccine Gudair® (Pfizer Animal Health, now Zoetis, Australia) in a single dose as recommended by the manufacturer. A negative control group comprised sheep that were not vaccinated. The treatment groups and vaccine formulations are described in Table 1.

**Table 1:** Treatment groups and vaccine formulations used in the trial.

Groups	Vaccine formulation	Adjuvant	Antigen
1S, 1D	50V	Montanide ISA 50V	Heat killed MAP 1 × 10^8^/dose
2S, 2D	50V2	Montanide ISA 50V2	Heat killed MAP 1 × 10^8^/dose
3S, 3D	61VG	Montanide ISA 61VG	Heat killed MAP 1 × 10^8^/dose
4S, 4D	70MVG	Montanide ISA 70 MVG	Heat killed MAP 1 × 10^8^/dose
5S, 5D	71VG	Montanide ISA 71VG	Heat killed MAP 1 × 10^8^/dose
6S, 6D	201VG	Montanide ISA 201VG	Heat killed MAP 1 × 10^8^/dose
7S, 7D	Gel01	Montanide Gel 01 PR	Heat killed MAP 1 × 10^8^/dose
8S, 8D	No adjuvant	Phosphate buffered saline	Heat killed MAP 1 × 10^8^/dose
9	Positive control - vaccine Gudair^®^	As supplied by the manufacturer	Killed MAP as supplied in the vaccine by the manufacturer
10	Negative control -unvaccinated	None	None

Vaccine formulations given to sheep in Groups 1 to 8 were tested both as single dose (S) and a double dose (D) (a primary dose followed 4 weeks later by a booster dose). All groups consisted of 5 sheep.

### 2.4 Adjuvants

Adjuvants from the Montanide^**TM**^ ISA (SEPPIC, France) series used in the novel vaccine formulations for this study included five W/O, a W/O/W and a polymeric gel. These were Montanide^**TM**^ ISA 50V (W/O), Montanide^**TM**^ ISA 50V2 (W/O), Montanide^**TM**^ ISA 61VG (W/O), Montanide^**TM**^ ISA 70 M VG (W/O), Montanide^**TM**^ ISA 71 VG (W/O), Montanide^**TM**^ ISA 201 VG (W/O/W) and Montanide^**TM**^ Gel 01 PR (Polymeric gel).

### 2.5 Vaccines

Eight vaccine formulations were used in this study, 1 = ISA 50V, 2 = 50V2, 3 = 61VG, 4 = 70M VG, 5 = 71VG, 6 = 201VG, 7 = Gel 01, 8 = No Adjuvant. The Gudair**®** vaccine comprised killed MAP (Strain 316f) cells in a mineral oil adjuvant as prepared by the manufacturer. A single dose of the novel formulations contained approximately 1 × 10^8^ organisms of MAP (S strain, Telford 9.2, heat killed at 70 °C for 2 hours). MAP inactivation was confirmed by liquid culture [14]. The antigen and adjuvant components were mixed at a ratio of 60:40 vol/vol (adjuvant:antigen) under aseptic conditions and emulsified by vortexing the mixture for 2 mins.

All novel vaccines were tested for sterility by aerobic culture on sheep blood agar incubated at 37 °C for 48 hours, prior to use.

### 2.6 Vaccination

The vaccines were administered by subcutaneous injection high on the neck, behind the ear as a 1 mL dose. All vaccines were given on the right side of the neck. At 4 weeks post primary administration, groups requiring a booster dose were given a second dose of the same vaccine formulation. Gudair**®** vaccine was administered only as a single dose, according to the manufacturer's instructions.

### 2.7 Collection of blood samples

Blood samples (9 mL) were collected by jugular venepuncture into tubes without anticoagulant from all animals immediately before vaccination and at 2, 3, 4, 5, 6, 7, 8, 10, 14, 18, 22 and 26 weeks post primary vaccination. The blood tubes were centrifuged at 1455 x *g* and serum was aspirated into screw-capped tubes. Blood samples for the IFN-γ assay were collected pre-vaccination and then monthly for 6 months by jugular venipuncture into vacuum collection tubes containing lithium heparin (Vacuette). Serum samples were stored at −20 °C until required while heparinised blood was held at room temperature (≤5 hr) prior to stimulation with antigens for the IFN-γ assay.

### 2.8 Assessment of injection site lesions

The site of injection was monitored weekly until 10 weeks post vaccination and then monthly until 6 months post vaccination. The area around the injection site was palpated and visually inspected for the presence of swelling, and open lesion or abscess formation. Injection site lesions were defined as having a diameter greater than 0.5 cm, measured in one axis. Smaller lesions were detected by palpation, but not frequently or consistently, and were therefore not included in the data set. Injection site lesion data are presented on a group basis for sheep in each treatment aggregated across all the observations.

### 2.9 Serological assay to measure antibodies specific to MAP

An indirect ELISA incorporating a complex MAP antigen was employed to detect MAP-specific antibody in serum [15]. Results were expressed as the mean optical density signal from two replicates.

### 2.10 IFN gamma assay (IFN-γ)

Heparinised blood (0.5 mL) was stimulated in a 48-well plate with 0.5 mL of mycobacterial purified protein derivative (PPD) antigen (Prionics) at 20 μg/mL. The negative control for each sample consisted of blood with 0.5 mL of culture medium while the positive control had 0.5 mL of media with pokeweed mitogen (PWM) (Sigma) added at 10 μg/mL. After 48 hr incubation at 37 °C in air supplemented with 5% CO_2,_ the culture supernatant was collected and stored at −20 °C. The ELISA was carried out and the OD data were converted to sample to positive percent (SP%) as described by Begg et al 2010 [13].

### 2.11 Statistical analysis

Descriptive analyses were initially conducted and included creation of frequency tables for categorical variables and calculation of summary statistics for quantitative variables. Incidence of injection site lesions was calculated as the proportion of animals in each group at the start of the trials that developed injection site lesions. Relative risk was calculated to compare incidence risk between different vaccine formulations, and the significance of differences in proportions was determined using Fisher's exact test followed by two-sided two sample binomial tests.

Sizes of injection site lesions between treatment groups were compared using the non-parametric Kruskal-Wallis test because the distribution of injection site lesions was skewed invalidating assumptions of parametric tests. Further pairwise two-sample Wilcoxon comparisons were made to compare lesion sizes between pairs of different vaccine formulations.

MAP-specific IFN-γ and antibody responses were compared between the different vaccine formulations using the linear mixed modelling approach by including IFN-γ and antibody responses as outcomes in their respective models: vaccine formulations, time and their interactions as fixed effects; and animals as a random effect to account for multiple observations for each animal. IFN-γ and antibody responses were log transformed to meet the assumption of normality and homoscedasticity of variance was evaluated using residual diagnostics.

Unless otherwise stated the analyses were conducted using the SAS statistical program (© 2002–2012 SAS Institute Inc., Cary, NC, USA). All p-values reported in the manuscript are two-sided.

## 3 Results

### 3.1 Injection site lesions

**Fig. 1:**
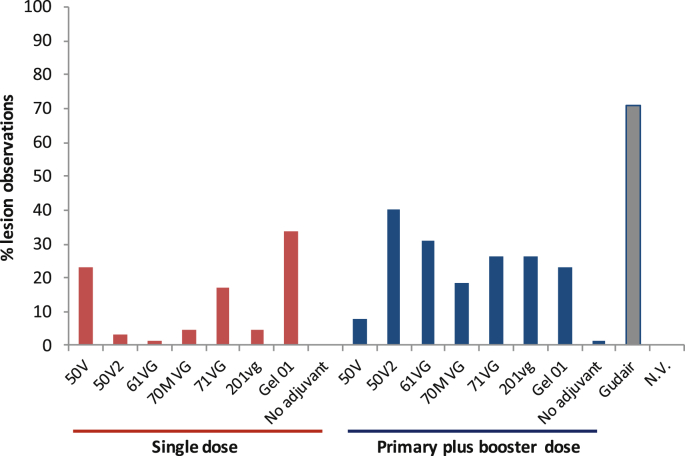
Percentage of occasions when injection site lesions were observed across all time points for the different vaccine formulations. Comparison of a single dose or double dose (booster given 1 month after the primary vaccination). Control groups were sheep given a single dose of Gudair®, and sheep that were not vaccinated (N.V.).

Sheep given a single dose of Gudair® vaccine developed injection site lesions that tended to be larger, persisted longer and were more common than in sheep given a single dose of most of the other formulations (Fig. 1 and Tables 2 and 3). The overall Fisher's exact test was significant (P < 0.05); for all groups except 50V, sheep given a single dose of Gudair® had a significantly greater probability (P < 0.05) of developing an injection site lesion than sheep given the other MAP vaccine formulations; the relative risk was 1.67–5 times for a single dose and 1.25 to 5 times for a double dose of the other formulations (Table 2). Sheep that were given two doses of the novel MAP vaccines were significantly more likely (relative risk: 2.8; 95% confidence interval 1.58, 4.97; *P* < 0.001) to develop an injection site lesion than animals that received only one dose (excluding animals given Gudair®) (Tables 2 and 3).

**Table 2:** Injection site lesions in sheep given a single dose of each vaccine formulation. There were no lesions in unvaccinated controls.

Vaccine formulation	No of animals observed with lesions/total number of animals in the group	Relative risk of lesion development (Gudair^®^ versus other vaccines^a^)	Number of lesion observations for the treatment group	Mean lesion size (cm)	Mean weeks to first recorded lesion	Mean weeks between first and last lesion observation
50V	3/5	1.67	15	1.3	2	19
50V2	1/5*	5.00	2	0.9	1	5
61VG	1/5*	5.00	1	0.8	3	1
70MVG	1/5*	5.00	3	1.1	2	24
71VG	1/5*	5.00	11	1.3	1	25
201VG	1/5*	5.00	3	2.0	8	6
Gel01	2/5*	2.50	22	1.4	1.5	24.5
No adjuvant	0/5	-	-	-	-	-
Gudair^**®**^	5/5	-	46	2.2	2.2	23.8

*P < 0.05 compared to Gudair.

a Relative risk for Gudair^**®**^ compared to other vaccines; animals given Gudair^**®**^ vaccine were 1.67–5 times more likely to develop lesions compared to those given other vaccines.

**Table 3:** Injection site lesions in sheep given two doses^a^ of each vaccine formulation. There were no lesions in unvaccinated controls.

Vaccine formulation	No of animals observed with lesions/total number of animals in the group	Relative risk of lesion development (Gudair^®^ versus other vaccines^b^)	Number of lesion observations for the treatment group	Mean lesions size (cm)	Mean weeks to first recorded lesion	Mean weeks between first and last lesion observation
50V	4/5	1.25	6	1.7	9	3
50V2	4/5	1.25	26	1.9	2.5	17
61VG	4/5	1.25	20	2.1	5.75	15.75
70MVG	3/5	1.67	12	2.2	6.0	10.7
71VG	3/5	1.67	17	2.0	5.7	14.7
201VG	5/5	1.00	17	1.5	9.6	12
Gel01	4/5	1.25	15	1.3	3.75	8.25
No adjuvant	1/5	5.00	1	1.2	3	1

a a primary dose followed 4 weeks later by a booster dose.

b Relative risk for a single dose of Gudair^**®**^ compared to a primary and a booster dose of the other vaccines; animals given Gudair^**®**^ vaccine were 1.25–5 times more likely to develop lesions compared to those given other vaccines.

### 3.2 MAP-specific IFN-γ and antibody responses

The IFN-γ responses of the sheep given different vaccine formulations were monitored over time (Tables 4 and 5). There were no significant differences between the IFN-γ response attributable to the number of doses of vaccine given for any of the formulations containing adjuvant. Vaccine formulations 70MVG and Gel01 did not stimulate an antigen-specific IFN-γ response after vaccination with either one or two doses and were not significantly different from the No adjuvant and unvaccinated groups. Gudair® vaccinated animals had a significantly greater (*P* < 0.05) antigen specific IFN-γ response than animals given the formulations 70MVG, Gel01, 201VG, 61VG, No adjuvant or those left unvaccinated. There was no significant difference between the IFN-γ response from the Gudair® vaccinated animals and sheep given the formulations 50V, 50V2 and 71VG.

**Table 4:** Mean antigen-specific IFN-γ responses from sheep vaccinated with a single dose of each vaccine formulation.

Vaccine formulation	Weeks post vaccination						
	0	4	8	14	18	22	26
50V	0.38 ± 0.3	3.91 ± 3.5	14.27 ± 12.8	57.81 ± 51.7	34.40 ± 30.7	18.01 ± 16.1	11.85 ± 11.5
50V2	0.50 ± 0.4	1.01 ± 0.9	11.93 ± 10.7	6.99 ± 6.3	6.19 ± 5.5	3.85 ± 3.4	2.80 ± 2.5
61VG	0.24 ± 0.2	1.28 ± 1.1	8.35 ± 8.2	4.08 ± 4.0	13.14 ± 12.9	15.90 ± 15.6	4.40 ± 4.3
70MVG	0.15 ± 0.1	0.60 ± 0.5	3.39 ± 3.0	2.66 ± 2.4	9.36 ± 8.4	4.30 ± 3.8	4.52 ± 4.0
71VG	0.70 ± 0.6	0.89 ± 0.8	2.33 ± 2.1	5.92 ± 5.3	7.70 ± 6.9	3.24 ± 2.9	2.71 ± 2.4
201VG	0.25 ± 0.2	1.08 ± 1.0	2.54 ± 2.3	0.95 ± 0.9	2.34 ± 2.1	2.65 ± 2.3	3.11 ± 2.8
Gel01	0.45 ± 0.4	0.20 ± 0.2	5.04 ± 4.5	5.84 ± 5.2	7.49 ± 6.7	1.43 ± 1.3	2.79 ± 2.5
No adjuvant	0.11 ± 0.1	0.38 ± 0.3	5.62 ± 5.0	0.30 ± 0.3	0.50 ± 0.5	1.24 ± 1.1	0.22 ± 0.2
Gudair^**®**^	0.67 ± 0.6	7.66 ± 6.8	44.91 ± 40.1	27.34 ± 24.4	23.21 ± 20.7	12.24 ± 10.9	19.22 ± 18.7
Unvaccinated	0.88 ± 0.8	0.61 ± 0.5	6.47 ± 5.8	0.21 ± 0.2	5.94 ± 5.3	0.87 ± 0.8	1.22 ± 1.1

Data shown: geometric mean SP% response ± s.e. from 5 animals in each treatment group.

**Table 5:** Mean antigen specific IFN-γ responses from sheep vaccinated with two doses^a^ of the formulations of killed MAP and different adjuvants.

Vaccine formulation	Weeks post primary vaccination						
	0	4	8	14	18	22	26
50V	0.45 ± 0.4	4.00 ± 3.9	12.90 ± 11.5	5.00 ± 4.5	10.08 ± 9.0	2.99 ± 2.7	2.77 ± 2.5
50V2	0.24 ± 0.2	1.24 ± 1.1	25.53 ± 22.8	13.42 ± 12.0	18.15 ± 16.2	8.47 ± 7.6	10.19 ± 9.1
61VG	0.31 ± 0.3	0.70 ± 0.6	6.39 ± 5.7	1.26 ± 1.1	8.85 ± 7.9	0.85 ± 0.8	4.22 ± 3.8
70MVG	0.92 ± 0.8	0.92 ± 0.8	2.28 ± 2.0	0.70 ± 0.7	5.46 ± 4.9	1.92 ± 1.7	0.93 ± 0.8
71VG	0.14 ± 0.1	0.19 ± 0.2	6.75 ± 6.0	31.45 ± 28.1	28.58 ± 25.5	14.07 ± 12.6	17.10 ± 15.3
201VG	0.27 ± 0.2	1.18 ± 1.1	10.63 ± 9.5	4.82 ± 4.3	26.85 ± 24.0	12.15 ± 10.9	14.67 ± 13.1
Gel01	0.25 ± 0.2	0.14 ± 0.1	1.34 ± 1.2	0.09 ± 0.1	2.27 ± 2.0	0.14 ± 0.1	0.14 ± 0.1
No adjuvant	0.16 ± 0.1	0.12 ± 0.11	7.36 ± 6.6	0.60 ± 0.6	4.98 ± 4.5	0.46 ± 0.4	2.07 ± 1.8
Unvaccinated	0.88 ± 0.8	0.61 ± 0.5	6.47 ± 5.8	0.21 ± 0.2	5.94 ± 5.3	0.87 ± 0.8	1.22 ± 1.1

Data shown: geometric mean SP% response ± s.e. from 5 animals in each treatment group.

a a primary dose followed 4 weeks later by a booster dose.

**Fig. 2:**
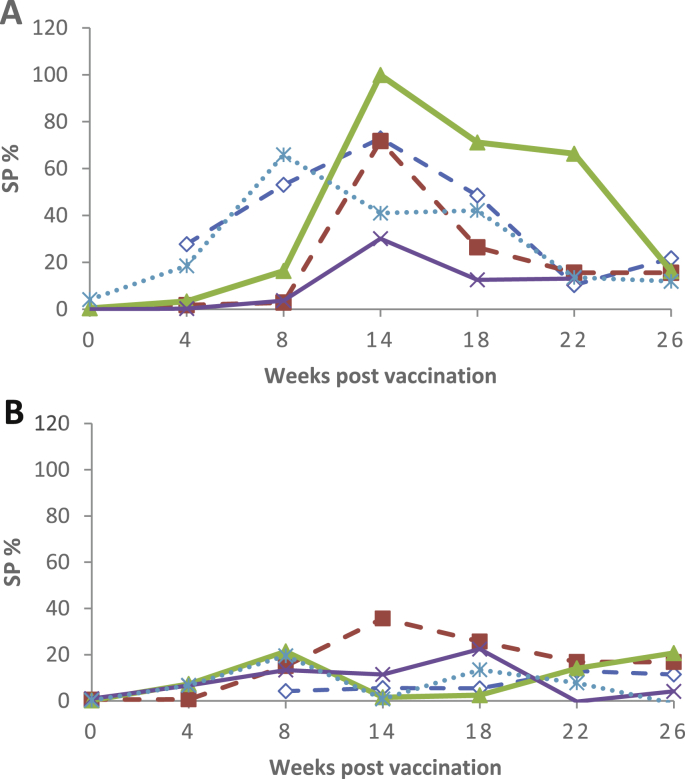
Antigen specific IFN-γ responses from the individual sheep given formulation 50V. A: Single dose. B: Primary plus booster dose. Each line represents data for an individual sheep.

Vaccine formulations 50V2, 71VG and 201VG showed a trend towards increased antigen-specific IFN-γ response compared to No adjuvant in sheep given a second dose of the same formulation four weeks after the primary dose (Tables 4 and 5). Vaccine formulations 50V and 61VG resulted in a trend towards lower antigen specific IFN-γ production after the booster vaccination compared to the group that were given a single dose. This was most evident for formulation 50V, with 4 of the 5 sheep that were given a booster dose having a lower antigen-specific IFN-γ response than the sheep given a single dose (Fig. 2).

The antigen-specific antibody levels were significantly greater (*P* < 0.001) in sera collected from Gudair® vaccinated sheep compared to sheep vaccinated with the other vaccine formulations (Tables 6 and 7). Animals that were given two doses of Gel 01, 201VG, 61VG, and 70M VG vaccines had increased (*P* < 0.05) serum antigen-specific antibody levels compared to sheep given a single dose of the same formulation. Sheep given a single dose of the formulations 61VG, 70M VG and 201VG produced low levels of specific antibody, similar to animals given no adjuvant. For the two vaccine formulations where the booster vaccination led to reduced IFN-γ responses, the antigen-specific antibody responses were similar (50V) or significantly increased (61VG, *P* < 0.005, weeks 6–10) compared to those seen in the single dose vaccinated sheep (Tables 6 and 7).

**Table 6:** Mean specific antibody responses from sheep vaccinated with a single dose of each vaccine formulation.

Vaccine formulation	Weeks post vaccination												
	0	2	3	4	5	6	7	8	10	14	18	22	26
50V	0.10 ± 0.02	0.17 ± 0.03	0.20 ± 0.04	0.41 ± 0.09	0.23 ± 0.05	0.20 ± 0.04	0.31 ± 0.06	0.31 ± 0.06	0.33 ± 0.07	0.32 ± 0.07	0.37 ± 0.08	0.41 ± 0.09	0.18 ± 0.04
50V2	0.11 ± 0.02	0.28 ± 0.06	0.34 ± 0.07	0.57 ± 0.12	0.36 ± 0.08	0.33 ± 0.07	0.37 ± 0.08	0.41 ± 0.09	0.41 ± 0.09	0.27 ± 0.06	0.33 ± 0.07	0.29 ± 0.06	0.19 ± 0.04
61VG	0.11 ± 0.02	0.13 ± 0.03	0.18 ± 0.04	0.28 ± 0.06	0.15 ± 0.03	0.13 ± 0.03	0.17 ± 0.04	0.14 ± 0.03	0.15 ± 0.04	0.18 ± 0.04	0.21 ± 0.05	0.24 ± 0.06	0.14 ± 0.03
70MVG	0.09 ± 0.02	0.12 ± 0.02	0.20 ± 0.04	0.30 ± 0.076	0.15 ± 0.03	0.11 ± 0.02	0.14 ± 0.03	0.14 ± 0.03	0.13 ± 0.03	0.11 ± 0.02	0.12 ± 0.03	0.14 ± 0.03	0.12 ± 0.03
71VG	0.10 ± 0.02	0.19 ± 0.04	0.25 ± 0.05	0.40 ± 0.08	0.23 ± 0.05	0.22 ± 0.05	0.28 ± 0.06	0.33 ± 0.07	0.41 ± 0.08	0.36 ± 0.08	0.35 ± 0.07	0.35 ± 0.07	0.22 ± 0.05
201VG	0.10 ± 0.02	0.22 ± 0.05	0.29 ± 0.06	0.37 ± 0.08	0.21 ± 0.04	0.21 ± 0.04	0.18 ± 0.04	0.27 ± 0.06	0.32 ± 0.07	0.27 ± 0.06	0.28 ± 0.06	0.26 ± 0.06	0.19 ± 0.04
Gel 01	0.11 ± 0.02	0.14 ± 0.03	0.21 ± 0.04	0.32 ± 0.07	0.17 ± 0.04	0.16 ± 0.03	0.19 ± 0.04	0.18 ± 0.04	0.18 ± 0.04	0.14 ± 0.03	0.18 ± 0.04	0.17 ± 0.04	0.11 ± 0.02
No adjuvant	0.13 ± 0.03	0.12 ± 0.03	0.18 ± 0.04	0.33 ± 0.07	0.16 ± 0.03	0.13 ± 0.03	0.15 ± 0.03	0.15 ± 0.03	0.15 ± 0.03	0.15 ± 0.03	0.16 ± 0.03	0.16 ± 0.03	0.12 ± 0.02
Gudair^**®**^	0.15 ± 0.03	0.30 ± 0.06	0.62 ± 0.13	1.14 ± 0.24	0.80 ± 0.17	0.68 ± 0.14	0.93 ± 0.19	0.92 ± 0.19	1.14 ± 0.24	0.53 ± 0.11	0.94 ± 0.20	0.85 ± 0.18	0.48 ± 0.10
Un-vaccinated	0.12 ± 0.03	0.09 ± 0.02	0.12 ± 0.02	0.23 ± 0.05	0.12 ± 0.03	0.11 ± 0.02	0.13 ± 0.03	0.12 ± 0.02	0.11 ± 0.02	0.09 ± 0.02	0.13 ± 0.03	0.12 ± 0.03	0.10 ± 0.02

Data shown: geometric mean OD response ± s.e. from 5 animals in each treatment group.

**Table 7:** Mean specific antibody responses from sheep vaccinated with two doses^a^ of each vaccine formulation.

Vaccine formulation	Weeks post primary vaccination												
	0	2	3	4	5	6	7	8	10	14	18	22	26
50V	0.10 ± 0.02	0.13 ± 0.03	0.16 ± 0.03	0.28 ± 0.06	0.20 ± 0.04	0.21 ± 0.04	0.27 ± 0.06	0.26 ± 0.05	0.28 ± 0.06	0.20 ± 0.04	0.29 ± 0.06	0.29 ± 0.06	0.16 ± 0.03
50V2	0.09 ± 0.02	0.19 ± 0.04	0.26 ± 0.05	0.37 ± 0.08	0.24 ± 0.05	0.52 ± 0.11	0.63 ± 0.13	0.63 ± 0.13	0.73 ± 0.15	0.45 ± 0.09	0.41 ± 0.09	0.40 ± 0.08	0.24 ± 0.05
61VG	0.10 ± 0.02	0.14 ± 0.03	0.18 ± 0.04	0.29 ± 0.06	0.20 ± 0.04	0.41 ± 0.09	0.43 ± 0.09	0.42 ± 0.09	0.45 ± 0.10	0.28 ± 0.06	0.33 ± 0.07	0.30 ± 0.06	0.23 ± 0.05
70MVG	0.10 ± 0.02	0.10 ± 0.02	0.14 ± 0.03	0.29 ± 0.06	0.13 ± 0.03	0.19 ± 0.04	0.20 ± 0.04	0.23 ± 0.05	0.24 ± 0.05	0.19 ± 0.04	0.23 ± 0.05	0.25 ± 0.05	0.19 ± 0.04
71VG	0.09 ± 0.02	0.17 ± 0.04	0.22 ± 0.05	0.34 ± 0.07	0.25 ± 0.05	0.37 ± 0.08	0.42 ± 0.09	0.42 ± 0.09	0.57 ± 0.12	0.44 ± 0.09	0.52 ± 0.11	0.52 ± 0.11	0.32 ± 0.07
201VG	0.11 ± 0.02	0.13 ± 0.03	0.24 ± 0.05	0.39 ± 0.08	0.36 ± 0.08	0.39 ± 0.08	0.37 ± 0.08	0.31 ± 0.06	0.33 ± 0.07	0.18 ± 0.04	0.24 ± 0.05	0.18 ± 0.04	0.17 ± 0.04
Gel01	0.09 ± 0.02	0.10 ± 0.02	0.19 ± 0.04	0.21 ± 0.04	0.26 ± 0.05	0.35 ± 0.07	0.42 ± 0.09	0.42 ± 0.09	0.42 ± 0.09	0.19 ± 0.04	0.17 ± 0.04	0.17 ± 0.04	0.17 ± 0.04
No adjuvant	0.11 ± 0.02	0.09 ± 0.02	0.13 ± 0.03	0.22 ± 0.05	0.12 ± 0.03	0.13 ± 0.03	0.15 ± 0.03	0.14 ± 0.03	0.13 ± 0.03	0.17 ± 0.04	0.15 ± 0.03	0.15 ± 0.03	0.11 ± 0.02

Data shown: geometric mean OD response ± s.e. from 5 animals in each treatment group.

a A primary dose followed 4 weeks later by a booster dose.

**Fig. 3:**
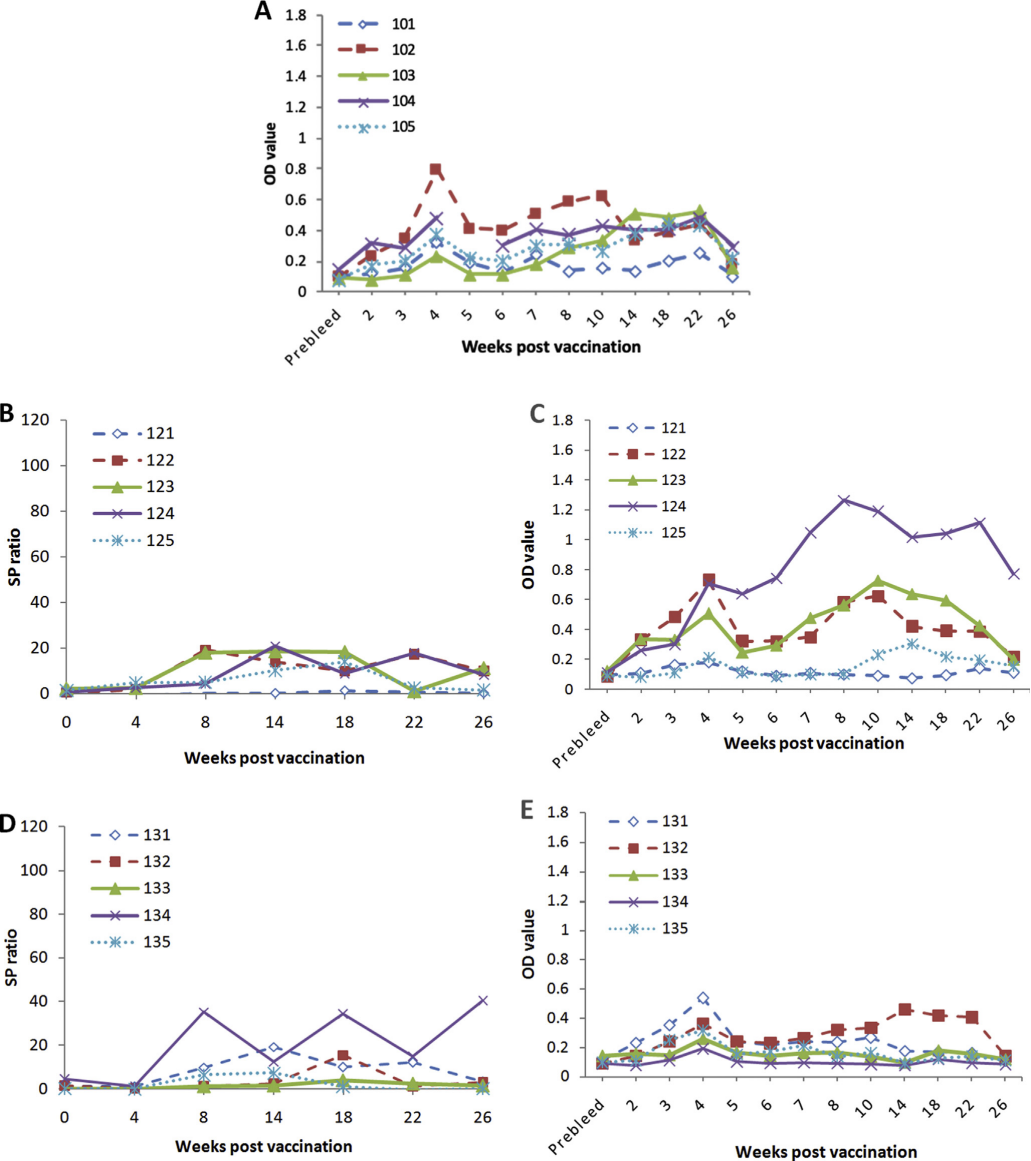
Antigen specific IFN-γ and antibody responses from individual animals given a single dose of formulations of killed MAP and various adjuvants that resulted in different immune profiles. A: Antibody responses from sheep given formulation 50V. B: IFN-γ responses from sheep given formulation 71VG. C: Antibody responses from sheep given formulation 71VG. D: IFN-γ responses from sheep given formulation Gel01. E: Antibody responses from sheep given formulation Gel01.

Overall, the results indicated that the immune response profile to heat-killed MAP antigen was altered by the adjuvant in the formulation. With no adjuvant, the heat-killed MAP did not induce a significant elevation in either the serum antibody or antigen-specific IFN-γ memory response compared to the unvaccinated sheep (Tables 4, 5, 6, and 7). Immune response patterns ranging from biased cell mediated to biased humoral immunity were found with different formulations. For example, an immune bias towards IFN-γ was generated using adjuvant 50V with a single dose (Figs. 2 A and 3A) and an antibody/humoral immune bias was seen when using adjuvant 71VG as a single dose (Fig. 3 B and C). The formulation comprising adjuvant 50V2 and heat-killed MAP given in 2 doses created a mixed response with elevated IFN-γ and antibody levels (Tables 5 and 7).

### 3.3 Association between injection site lesions and immune response

The development of injection site lesions was not always associated with a strong immune response. Of the sheep vaccinated with a single dose of killed MAP with adjuvant Gel 01, injection site lesions were observed at 33% of the recordings, predominantly in 2 sheep. These two sheep (131 and 132) had low levels of antigen-specific IFN-γ and antibodies (Fig. 3 D and E) but accounted for 23–33% of injection site lesions across all observations.

## 4 Discussion

We have demonstrated that immunisation of sheep with formulations comprising heat killed MAP and different adjuvants results in different immunological profiles. The immune response was altered also by the use of a second (booster) dose of the same vaccine; in some cases this resulted in a lower cell mediated immune response compared to a single dose.

Testing the immunogenicity of a mycobacterial antigen with different adjuvants is logical, especially with regard to recombinant antigens [12]. In this study, the testing of highly refined mineral oil adjuvants with a complex whole cell mycobacterial antigen led to a range of unexpected results. The theoretical optimal immune profile proposed for protection against mycobacterial infections including MAP is a cell mediated/IFN-γ biased response [16, 17, 18, 19, 20]. The commercially available MAP vaccines provide incomplete protection against JD, but results in a strong mixed cellular IFN-γ and humoral immune response [2, 10]. Others are examining how to best develop vaccines with a bias towards a Th1/IFN-γ response [16]. This study has shown that by altering the adjuvant, different immunological profiles can be achieved ranging from cellular IFN-γ, humoral, or mixed responses. Such widely differing immune responses to the same antigen have not previously been observed in JD vaccine development, probably due to the limited number of adjuvants that were tested previously [8, 9]. This finding has significance for JD vaccine development: novel antigens should be tested with a wider range of adjuvants. Furthermore, previously tested poorly immunogenic antigens may need to be re-examined to determine whether a preferred immune profile and possible protection can be established using different mineral oil adjuvants.

The use of a single or double (booster) dose of the formulations also resulted in unexpected alterations to the immune profile for some of the novel vaccines. Typically, only one dose of mycobacterial vaccine is administered to ruminants, for example Gudair® in sheep. Other types of inactivated vaccines are given to sheep but typically these require a primary dose and a booster dose to achieve optimal immune responses (for example see [21]). In this study, giving a second dose of formulations 50V and 61VG did not boost the immune response as expected but resulted in a reduced IFN-γ response. This may be due to a negative feedback loop, via release of Immunoregulatory cytokines such as IL-10 or IL-4, or preferential activation of T regulatory cell subsets rather than effector cells. The concomitant activation of T regulatory and effector cell phenotypes and expression of the immune checkpoint molecule Programmed cell death protein 1 (PD-1) by antigen-specific CD4^+^ T cell populations has been seen post-vaccination in other mycobacterial diseases [22]. A detailed assessment of MAP-specific CD4^+^ T cell populations, including cytokine and cell surface markers, is required to conclusively determine the mechanism of this post-booster effect.

While the second dose of 61VG resulted in a slight increase in antibody response, no difference was seen in the antibody response of sheep given a booster dose of formulation 50V. This indicates either that the immune response may have been inhibited by a second dose of vaccine but further examination was beyond the scope of this study.

All of the vaccines tested in this study resulted in fewer injection site lesion scores compared to Gudair®. It is thought that the injection site lesions associated with Gudair® and other killed MAP mineral oil vaccines are due to the Freund's-like nature of the vaccines [5]. The use of highly refined mineral oils and emulsification protocols is the most probable reason for the reduced injection site lesions in this study. Another possible explanation for the reduced injection site lesions could be a disparity in the number of killed MAP in the formulations, however this cannot be confirmed as the number of killed MAP in Gudair® is not disclosed. A new commercial MAP vaccine, Silirium®, uses highly refined mineral oils in the adjuvant with the aim to develop fewer injection site lesions, however this vaccine is also not fully protective [3, 23].

Vaccination site lesions are considered to be due to the interaction between the adjuvant, the antigen and the immune response of the host. However, for a number of formulations, lesions were found in animals or groups with a low systemic immune response. One of the adjuvants, Montanide Gel 01, a polymeric (sodium polyacrylate) gel, resulted in injection site lesions but the acquired immune response was negligible. This raises the possibility that this formulation induces an inflammatory response that is not specific to the antigen. It is possible that there were significant immune responses, not measured in this study (for example pro-inflammatory cytokines) that may have effected lesion formation. Caution must also be taken when interpreting these results, as the trial has not been replicated.

This study indicates that the adjuvant mixed with killed MAP influences the immune response and the incidence of injection site lesions. Although we did not investigate the effect of the strain of MAP, others have shown that this can make a difference to the immune response and pathology that develops during an active MAP infection [24]. Currently the critical parts of the protective immune response induced by commercial mycobacterial vaccines are unknown [20] and it may now be possible to uncouple protective immunity from excessive tissue reactivity. With this knowledge it may also be possible to formulate and test the efficacy of vaccines that produce targeted immunological profiles suited to protection against other pathogens, i.e. those for which a bias towards cellular or humoral immunity would be advantageous based on understanding of pathogenesis.

## Declarations

### Author contribution statement

D J Begg: Conceived and designed the experiments; Performed the experiments; Analyzed and interpreted the data; Contributed reagents, materials, analysis tools or data; Wrote the paper.

O Dhungye: Conceived and designed the experiments; Contributed reagents, materials, analysis tools or data.

A Naddi: Performed the experiments; Analyzed and interpreted the data; Contributed reagents, materials, analysis tools or data.

N K Dhand: Analyzed and interpreted the data.

K M Plain: Conceived and designed the experiments; Contributed reagents, materials, analysis tools or data.

K de Silva: Conceived and designed the experiments; Analyzed and interpreted the data; Contributed reagents, materials, analysis tools or data.

A C Purdie: Conceived and designed the experiments.

R J Whittington: Conceived and designed the experiments; Analyzed and interpreted the data; Wrote the paper.

### Funding statement

This work was supported by Meat and Livestock Australia and by Cattle Council of Australia, Sheepmeat Council of Australia and Wool Producers Australia through Animal Health Australia.

### Competing interest statement

The authors declare no conflict of interest.

### Additional information

No additional information is available for this paper.

## References

[1] Bush R.D., Windsor P.A., Toribio J.A. (2006). Losses of adult sheep due to ovine Johne's disease in 12 infected flocks over a 3-year period. Aust. Vet. J..

[2] Reddacliff L., Eppleston J., Windsor P., Whittington R., Jones S. (2006). Efficacy of a killed vaccine for the control of paratuberculosis in Australian sheep flocks. Vet. Microbiol..

[3] Rosseels V., Huygen K. (2008). Vaccination against paratuberculosis. Expert Rev. Vaccines.

[4] Eppleston J., Windsor P.A. (2007). Lesions attributed to vaccination of sheep with Gudair for the control of ovine paratuberculosis: post farm economic impacts at slaughter. Aust. Vet. J..

[5] Windsor P.A., Bush R., Links I., Eppleston J. (2005). Injury caused by self-inoculation with a vaccine of a Freund's complete adjuvant nature (Gudair) used for control of ovine paratuberculosis. Aust. Vet. J..

[6] Aucouturier J., Ascarateil S., Dupuis L. (2006). The use of oil adjuvants in therapeutic vaccines. Vaccine.

[7] Windsor P.A., Eppleston J. (2006). Lesions in sheep following administration of a vaccine of a Freund's complete adjuvant nature used in the control of ovine paratuberculosis. N. Z. Vet. J..

[8] Kathaperumal K., Park S.U., McDonough S., Stehman S., Akey B., Huntley J. (2008). Vaccination with recombinant Mycobacterium avium subsp. paratuberculosis proteins induces differential immune responses and protects calves against infection by oral challenge. Vaccine.

[9] Santema W., Hensen S., Rutten V., Koets A. (2009). Heat shock protein 70 subunit vaccination against bovine paratuberculosis does not interfere with current immunodiagnostic assays for bovine tuberculosis. Vaccine.

[10] Begg D.J., Griffin J.F. (2005). Vaccination of sheep against *M. paratuberculosis*: immune parameters and protective efficacy. Vaccine.

[11] Hines M.E., Stiver S., Giri D., Whittington L., Watson C., Johnson J. (2007). Efficacy of spheroplastic and cell-wall competent vaccines for Mycobacterium avium subsp. paratuberculosis in experimentally-challenged baby goats. Vet. Microbiol..

[12] Vordermeier H.M., Dean G.S., Rosenkrands I., Agger E.M., Andersen P., Kaveh D.A. (2009). Adjuvants induce distinct immunological phenotypes in a bovine tuberculosis vaccine model. Clin. Vaccine Immunol..

[13] Begg D.J., de Silva K., Di Fiore L., Taylor D.L., Bower K., Zhong L. (2010). Experimental infection model for Johne's disease using a lyophilised, pure culture, seedstock of Mycobacterium avium subspecies paratuberculosis. Vet. Microbiol..

[14] Whittington R.J., Marsh I., McAllister S., Turner M.J., Marshall D.J., Fraser C.A. (1999). Evaluation of modified BACTEC 12B radiometric medium and solid media for culture of Mycobacterium avium subsp. paratuberculosis from sheep. J. Clin. Microbiol..

[15] Gurung R.B., Begg D.J., Purdie A.C., Eamens G.J., Whittington R.J. (2015). Development of 316v antibody enzyme-linked immunosorbent assay for detection of paratuberculosis in sheep. Rev. Sci. Tech..

[16] Santema W., Rutten V., Koets A. (2011). Bovine paratuberculosis: recent advances in vaccine development. Vet. Q..

[17] Stabel J.R. (2006). Host responses to Mycobacterium avium subsp. paratuberculosis: a complex arsenal. Anim. Health Res. Rev..

[18] Goldsack L., Kirman J.R. (2007). Half-truths and selective memory: interferon gamma, CD4(+) T cells and protective memory against tuberculosis. Tuberculosis (Edinb).

[19] Thakur A., Pedersen L.E., Jungersen G. (2012). Immune markers and correlates of protection for vaccine induced immune responses. Vaccine.

[20] Andersen P., Woodworth J.S. (2014). Tuberculosis vaccines - rethinking the current paradigm. Trends Immunol..

[21] Dhungyel O.P., Whittington R.J. (2009). Modulation of inter-vaccination interval to avoid antigenic competition in multivalent footrot (Dichelobacter nodosus) vaccines in sheep. Vaccine.

[22] Day C., Tameris M., Mansoor N., van Rooyen M., de Kock M., Geldenhuys H. (2013). Induction and regulation of T-cell immunity by the novel tuberculosis vaccine M72/AS01 in South African adults. Am. J. Respir. Crit. Care Med..

[23] Alonso-Hearn M., Molina E., Geijo M., Vazquez P., Sevilla I.A., Garrido J.M. (2012). Immunization of adult dairy cattle with a new heat-killed vaccine is associated with longer productive life prior to cows being sent to slaughter with suspected paratuberculosis. J. Dairy Sci..

[24] Fernández M., Benavides J., Sevilla I.A., Fuertes M., Castaño P., Delgado L. (2014). Experimental infection of lambs with C and S-type strains of *Mycobacterium avium* subspecies *paratuberculosis*: immunological and pathological findings. Vet. Res..

